# Phylogenetic and phylodynamic approaches to understanding the evolution of non-small cell lung cancer using single-cell sequencing

**DOI:** 10.1038/s41388-026-03997-1

**Published:** 2026-09-24

**Authors:** Teng Li, Xianghan Li, Yiran Zou, Thomas K. F. Wong, Michael J. Campa, Elizabeth B. Gottlin, Edward F. Patz, Allen Rodrigo

**Affiliations:** 1 School of Biological Sciences, University of Auckland, Auckland, New Zealand; 2 Research School of Biology, Australian National University, Canberra, ACT Australia; 3 School of Computing, Australian National University, Canberra, ACT Australia; 4 Department of Radiology, Duke University Medical Center, Durham, NC USA; 5 Duke Cancer Institute, Duke University Medical Center, Durham, NC USA; 6 Department of Pharmacology and Cancer Biology, Duke University Medical Center, Durham, NC USA

**Keywords:** Non-small-cell lung cancer, Cancer genomics

## Abstract

Characterizing the genealogy of tumor cells and their population dynamics is central to our understanding of the evolution of cancer. While direct monitoring of the tumor evolutionary process is impractical, we leverage advances in phylogenetics and phylodynamics to explore the evolutionary dynamics of non-small cell lung cancer (NSCLC) using single-cell whole exome sequencing data from 351 tumor cells across 11 NSCLC patients, predominantly focusing on the primary tumors and early stages of NSCLC. Our results reveal distinct phylogenetic tree shapes and evolutionary trajectories across different stages of NSCLC evolution. However, this intricate and multi-phase evolutionary process cannot be fully elucidated by current tumor models. Herein, we hypothesize that tumors progress through the following phases: (1) an initial rapid expansion through Big Bang-like growth, (2) a prolonged plateau period of non-progression with possible accumulation of neutral substitutions, (3) a subsequent phase where adaptive substitutions accumulate gradually, (4) a bottleneck event where selectively advantageous subclones acquire invasive capabilities (characterized by a pattern of punctuated evolution), and, potentially, a rapid expansion leading to metastasis. This work highlights the use of single-cell phylogenetics and phylodynamics for interpreting the evolution of NSCLC, with potential in predicting cancer progression, resistance mechanisms, and treatment response.

## Introduction

Cancer currently ranks as the leading cause of death worldwide [1]. While lung cancer is the second most common cancer after breast cancer, it remains the leading cause of cancer-related mortality [2]. Non-small cell lung cancer (NSCLC) is the most common histological type and accounts for about 85% of all lung cancers, which includes adenocarcinoma, squamous cell carcinoma, large cell carcinoma, and other subtypes [3]. The overall 5-year survival rate of NSCLC is only 28%, which most likely reflects the fact that approximately 70% of newly diagnosed NSCLC patients have advanced-stage disease at presentation, resulting in a poor prognosis [4]. Hence, understanding and characterizing the trajectory of NSCLC progression is crucial to optimizing treatment and improving outcomes.

**Fig. 1:**
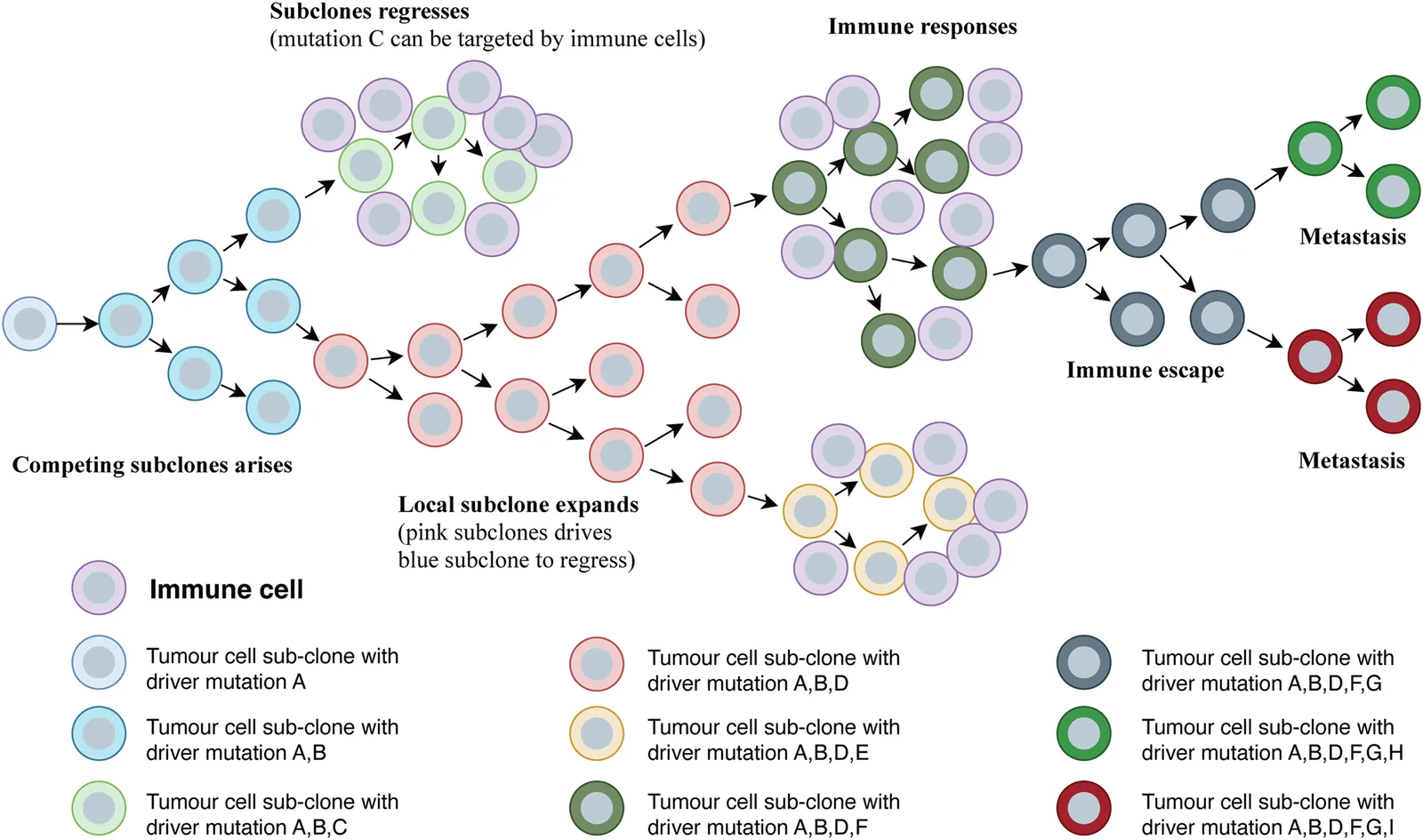
The branching evolution model, adapted from Nowell (1976). Subclonal tumor cells with different driver mutations progress over time, and each differently colored circle represents a genetically distinct subclone. Under selective pressures, such as immune responses, certain mutant subclones expand while others become extinct, regress, or remain dormant. Other selective pressures may also contribute to clonal expansion or extinction; these include increased reproductive potential and greater propensity to acquire resources or nutrients.

Tumor development was first depicted by Nowell as an evolutionary process that usually spans multiple decades [5]. According to this theory, a tumor initiates from a single normal cell and evolves in response to various selective pressures [6] (e.g., competition for space, nutrients, and other resources within a tumor; or differential recognition by the immune system allowing some cells to escape detection [7]) (Fig. 1). Since mutations accumulate during cell proliferation, genetic heterogeneity is expected to be present among all tumor cells [8, 9]. Some malignant cells originating from the primary tumor will consume available resources, become invasive, and spread to other parts of the body via the circulatory and lymphatic systems [10, 11]. Spread to other organs has a fitness cost to the tumor cells but is necessary for them to survive and proliferate. Evidence suggests that disseminated tumor cells may have either single (monoclonal) or multiple (polyclonal) origins [12]. There are also several competing models that describe the initiation and progression of cancer, including gradualism, the Big Bang model [13], and punctuated evolution [14] (Box 1), each of which may manifest in different tumors or at different stages of tumor evolution.

Recent large-scale multi-region and longitudinal studies have substantially advanced our understanding of NSCLC evolution [15–19]. In particular, several TRACERx (TRAcking non-small cell lung Cancer Evolution through therapy (Rx)) studies have established key principles of NSCLC evolution, including early clonal diversification [15], pervasive intratumor heterogeneity (ITH) [20], subclonal selection [18], immune-mediated selection [17], and branched metastatic seeding [15], all of which are associated with relapse and clinical outcome. In the TRACERx 421 cohort, large recent subclonal expansions were associated with positive subclonal selection and shorter disease-free survival [18]. Moreover, a companion TRACERx metastasis study demonstrated that metastatic dissemination in NSCLC can occur from the primary tumor through both monoclonal and polyclonal routes, with metastatic seeding subclones undergoing expansion and polyclonal dissemination associated with extrathoracic recurrence [15].

However, most of these studies relied on bulk multi-region sequencing, which infers clonal architecture from mixed cell populations and cannot directly reconstruct the genealogy of individual tumor cells. In contrast, single-cell sequencing technology has emerged as a novel approach in resolving ITH and understanding the evolution of cell lineages during tumorigenesis [21]. However, this method has its own challenges, including elevated amplification error rates, allelic dropout events, and uneven coverage depth, which are introduced by whole-genome amplification (WGA) (e.g., multiple displacement amplification (MDA) [11]) due to the limited amount of DNA present in a cell [22]. Moreover, copy number alterations typically cannot be analyzed due to the random priming bias introduced by MDA, which is difficult to correct systematically [23]. Fortunately, several innovative single-nucleotide variant (SNV) callers have been proposed to address these challenges associated with single-cell data, such as Monovar [24], SCcaller [25], SCIΦ [26], and ProSolo [27].

Single-cell phylogenetics aims to elucidate the genealogical relationships among cells within the same or different tumor regions [9, 28], where the branch lengths represent the evolutionary rates of the cell lineages [29, 30]. Phylodynamics in cancer focuses on estimating the dynamics of tumor populations and elucidates the evolutionary processes underpinning tumor progression by extracting biological insights from tumor phylogenies [31, 32]. Moreover, two important features of the tree shapes, the relative length of internal versus external branches and tree balance (Box 2), can provide additional evolutionary information about the changes in population size and selection pressure, respectively [33, 34].

Although these phylogenetic features were originally proposed for virus epidemiology, tumor cells have many traits in common with infectious agents, including higher-than-background mutation rates and the challenge of, and response to, immune selection [6, 32]. Thus, it is reasonable to extend the application of these phylodynamic approaches to individual tumor cells with the theory that this may reflect phenotype. Further, several novel approaches have been developed for inferring single-cell phylogenies from SNVs, using error models to correct specific single-cell WGA errors mentioned above and false-positive SNV calls. OncoNEM [35], SCITE [29], and SiFit [36] were based on the binary genotype matrix regardless of the nucleotides involved, while CellPhy [37] proposed a state-of-the-art error model considering the nucleotide substitution of diploid genotypes under a maximum likelihood framework. Additionally, Phylonco [38] implemented the CellPhy error model for diploid nucleotide genotypes within a Bayesian framework, and BEAST2 [39] which enables the inference of single-cell population dynamics using the Bayesian skyline plot [40].

In this study, we performed whole-exome sequencing (WES) on both single cells and bulk samples predominantly from stage IA through IIB primary tumors and matched normal tissues, respectively, from 11 NSCLC patients. Understanding the evolutionary dynamics of human cancer is quite challenging, since it is impractical to collect data from multiple time points for different stages within the same patient throughout tumor progression [41, 42]. Many tumors, including NSCLCs, are typically not detected at the earliest stages and, when they are detected, are often surgically resected soon after diagnosis [43, 44], thus precluding the possibility of studying tumor evolution in situ. We aim to employ SNV-based phylogenetic and phylodynamic approaches to explore the complex dynamics of NSCLC evolution and the evolutionary mechanisms behind metastatic dissemination based on single-cell WES data from different stages of NSCLC tumors and by leveraging these robust single-cell SNV callers and innovative probabilistic models for single-cell diploid genotypes. Our results suggest distinct evolutionary dynamics at different stages of tumor evolution; therefore, we propose a hypothesis-generating model for NSCLC evolution, which offers a more nuanced understanding of NSCLC progression and highlights the potential of single-cell phylogenetics and phylodynamics in predicting cancer progression and treatment outcomes.

Box 1: definitions Whereas cancer has long been recognized as an evolutionary process, cancer biologists sometimes use evolutionary terms that may not be commonly used in the evolutionary biology literature, or that may be used in ways that have subtle differences in meaning. In this section, we offer explicit definitions for these terms (cf. Davis et al. [41] and Cross et al. [14]). Before we begin, though, it is worth noting that the terms we define below can sometimes refer to both evolutionary patterns and evolutionary processes. Because it is possible for the same evolutionary pattern to emerge through quite different processes, it is sometimes difficult to discern whether the term is used to refer to a pattern or to a process. Here, we attempt to disentangle pattern from process where possible.

**Mutation and selection**

**Driver mutation** is a commonly used term in cancer biology and refers to mutations that confer a fitness advantage to a cell. Evolutionary biologists might refer to such mutations as adaptive mutations. **Passenger mutations** are mutations that have no fitness consequences. These are typically referred to as neutral mutations in the evolutionary biology literature. The terms “driver” and “passenger” appear to analogously convey the sense that driver mutations bring passenger mutations along for the ride. In those instances when this is what is meant, evolutionary biologists typically call this phenomenon “hitch-hiking”.

**Fitness** refers to the ability of an individual cell or variant to survive and reproduce; the term can also be used as a proxy for the average number of offspring a cell or variant can be expected to leave in the next generation. If different variants have different levels of fitness, then those with higher fitness will, on average, leave more offspring in succeeding generations.

**Genetic Drift** refers to the process where the frequencies of genetic variants change over time simply as a consequence of random sampling effects alone. With genetic drift, variants can go to fixation (i.e., all individuals in the population have that particular allele or mutation) or extinction (i.e., the variant is no longer present in the population).

**Selective sweep** is the process whereby a variant or mutation rapidly increases in relative frequency in a population, displacing other variants, because of a strong fitness advantage. After a selective sweep, the dominant variant is fixed in the population, and subsequent mutations may have relatively small or no fitness effects (i.e., they are nearly neutral or neutral, respectively).

**Selection** describes a suite of processes whereby mutations or traits that confer fitness benefits or disadvantages increase or decrease, respectively, in frequency in the population. Evolutionary biologists distinguish different types of selection, depending on the types of adaptations selected for. **Directional selection** refers to selection that acts on mutations/traits that provide or contribute to a particular phenotype within the phenotypic range; if the optimum phenotype is quantifiable, it is often closer to the tail end of a distribution. In contrast, **stabilizing selection** refers to selection that favors non-extreme (typically intermediate) phenotypes. **Diversifying selection** refers to processes that select for increased variation, and that typically result in a range of phenotypes or phenotypic values (e.g., individuals with genomic variants that cannot be recognized by the immune system or are able to evade the actions of drugs).

**Models of evolution**

**Linear evolution** describes both a pattern and a process whereby evolution proceeds through the replacement of one dominant variant in a population with another over time, through natural selection. Whereas it is possible that selective processes give rise to a linear pattern of evolution; it is equally possible that if the population is sufficiently small, mutations can become fixed sequentially in the population through genetic drift. It is important to note, therefore, that a pattern of apparent sequential (and “linear”) replacement of mutations is not always a consequence of a selective sweep (defined above). In this paper, and for consistency, we will use the term “linear evolution” in the same way that it is used in the cancer literature, and assume that replacement is mediated by selection.

**Gradualism** describes a process where small adaptive mutations accumulate gradually over time (perhaps at a consistent evolutionary tempo). Darwin envisaged adaptation through natural selection as a gradual process.

**Branching evolution** in cancer refers to the continuous divergence of tumor cell populations over time, driven by cell division and mutation. This process leads to the formation of distinct cell lineages within a tumor, where some lineages may expand, while others may become extinct or remain dormant. Each branch or lineage may accumulate neutral or selective mutations, although in the cancer literature, it is often assumed that lineages persist through the selection of driver ( = adaptive) mutations. It is possible, though, for lineages to persist for long periods of time through neutral evolution (see below), particularly when other lineages have not produced variants with sufficiently high fitness necessary to overcome the effects of stochastic sampling.

**Neutral evolution** describes the process whereby mutations that confer no fitness benefit or disadvantage (i.e., passenger mutations) arise randomly and subsequently become fixed substitutions or go to extinction. Neutral evolution is closely allied to genetic drift: genetic drift acts on the frequencies of mutations that have no selective effects (i.e., neutral mutations).

In the cancer literature [14, 41], **punctuated evolution** describes the process whereby mutations that facilitate tumor progression or metastases are acquired in short bursts (often at the beginning or during tumor formation) rather than gradually over time, interspersed with relatively long periods of stasis. The term is borrowed from the pattern first described by Eldredge and Gould [98, 99] whereby the fossil record shows long periods with little morphological change, followed by apparently rapid bursts of change.

**The Big Bang model** describes tumor growth as a single expansion where numerous intermixed subclones emerge early without stringent selection throughout growth, leading to a high level of heterogeneity [13]. Selection plays a minimal role after cancer initiation, allowing for the coexistence of multiple major clones, with driver mutations often being ancestral, characterizing a specific form of punctuated evolution with balanced phylogenetic trees.

**Methods of analysis**

**Phylogenetics** is the science of inferring the patterns of relationships of evolutionary units, i.e., biological entities (e.g., cells, organisms, species, etc.) that are part of ancestor-descendant relationships depicted as branching evolutionary trees or phylogenies.

**Phylodynamics** is a term that describes the use of evolutionary trees ( = phylogenies) to infer how populations change in size, and the intrinsic and extrinsic factors that act to mediate those changes. Inferences are made based on the shape of the trees (i.e., their topology or branching pattern), and the lengths of branches (or equivalently, the distances between nodes on the trees).

**Effective population size (**${{{\boldsymbol{N}}}}_{{{\boldsymbol{e}}}}$**)**: reflects the number of individuals that contribute offspring to the descendant generation; it is a population genetic parameter that can be a proxy measure of genetic heterogeneity. Technically, ${N}_{e}$ is the size of an ideal Wright-Fisher population [100] that accumulates genetic diversity at the same rate as the population under study. In phylodynamics, ${N}_{e}$ is often visualized over time via a **skyline plot**, i.e., a (smoothed) stepwise plot of ${N}_{e}$ at different levels of a phylogenetic tree. ${N}_{e}$ influences the extent to which genetic drift and selection play a role in the fixation of neutral or adaptive mutations: a small ${N}_{e}$ means that genetic drift can overwhelm selection, and adaptive mutations may have the same chance of survival as neutral mutations. In practice, changes in ${N}_{e}$ reflect changes in genetic diversity, and these changes may be due to many different factors including selective sweeps, population bottlenecks, clonal expansion, and so on.

Box 2: tree shapes Branches on phylogenetic trees can be classified into internal and external (or pendant) branches. Internal branches are branches that connect two ancestral nodes on a tree, whereas external branches connect an ancestral node to a tip of the tree (representing one sampled/observed individual in the dataset). External branches that are longer relative to internal branches indicate exponential growth in population size (A) under a coalescent model, whereas external branches that are shorter relative to internal branches reflect a constant population size (B) [101]. Meanwhile, the ladder-like phylogeny (imbalanced tree) may result from a population passing through strong selective pressure (i.e., selective sweeps), while the more balanced phylogeny suggests an absence of strong selection (C) [34].

**Figure:**
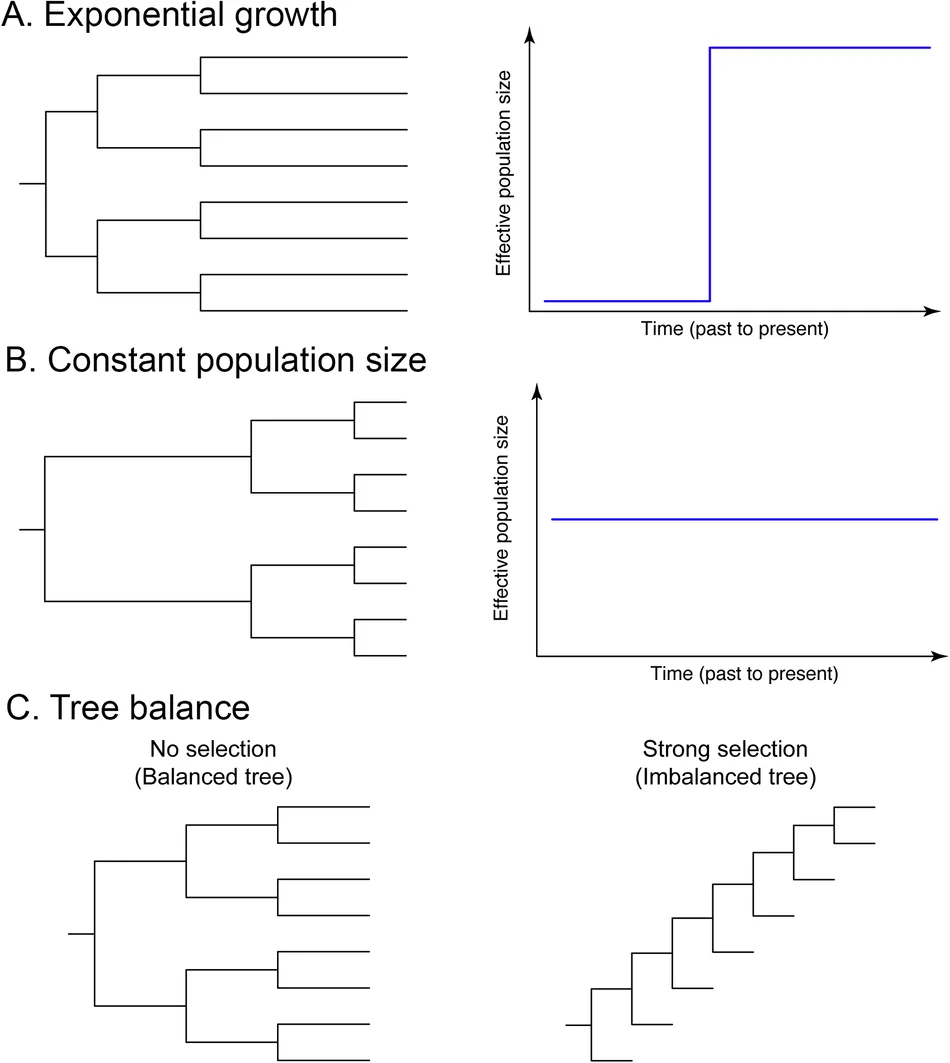

## Results and discussion

### Clinical summary of the patient and sample overview

We collected tumor and normal samples from 11 patients with NSCLC in stages IA through IIB (4 patients in IA, 3 patients in IB, 1 patient in IIA, and 2 patients in IIB), except Patient 17029, whose tumor was classified as stage IV (metastasis to spine) with a rare type of spindle cell carcinoma based on the histologic analysis (Table 1). Moreover, both patients with stage IIB had lymph node metastasis, which has prognostic value [45]. A total of 351 single-cell samples were isolated from the primary tumors of these 11 NSCLC patients (with a median of 23 cells per patient, ranging from 7 to 71) (Fig. S1), coupled with one corresponding normal bulk tissue for each patient (Table 1). Whole exome sequencing was performed for all the samples on the Illumina platform, with an average coverage depth of 198.1X for normal bulk tissues (median depth of 163.8 X) and 101.5X for tumor single cells (median depth of 100.1X) (Table S1).

**Table 1:** Demographics, cancer description, and tumor-processing information for 11 non-small cell lung cancer (NSCLC) patients.

Patient ID	Sex	Age at Dx	Stage at Dx	Histology	Status^a^	Tumor cells	Filtered cells	Normal Bulk	Filtered SNVs
16011	F	46	IB	AC	NM for 45 months	18	17	1	5018
17004	F	78	IA	SCC	NM for 40 months	71	66	1	5539
17005	M	76	IA	SCC	NM for 32 months	27	26	1	5107
17008	F	56	IA	AC	NM for 18 months	23	22	1	7027
17011	M	69	IB	AC	NM for 14 months	20	14	1	4089
17012	M	80	IIA	AC	NM for 15 months	23	23	1	6079
17017	F	66	IIB	LCC	Metastasis to lymph nodes, now deceased	46	37	1	4819
17028	F	72	IA	AC	NM for 27 months	59	50	1	3286
17029	M	64	IV	SpCC	Metastasis to spine, now deceased	47	38	1	3510
17030	M	61	IB	AC	NM for 36 months	7	7	1	1571
18001	F	79	IIB	AC	Metastasis to lymph nodes	10	9	1	2266

*Dx* diagnosis, *M* male, *F* female, *AC* adenocarcinoma, *SCC* squamous cell carcinoma, *LCC* large cell carcinoma, *SpCC* spindle cell carcinoma, *NM* non-metastatic NSCLC.

Information for these patients, previously described in Li et al. [68], has been updated with additional information from single-cell WES sequencing data and related analysis.

^a^Recurrence-free duration for the non-metastatic patients is as of their last clinic visit.

### SNV identification

After retaining only the SNVs identified by both robust single-cell callers (Monovar [24] and SCIΦ [26]) in tumor single cells, we identified a median of 4819 SNVs per patient (range from 1571 to 7027) that passed filtering criteria for subsequent analysis (Fig. S1 and Table 1). Moreover, a large proportion of detected SNVs in each patient (ranging from 57.1% to 68.6%) were present in the COSMIC database (Fig. S1). We also observed extensive ITH within each patient (Fig. S2), reflecting the complex evolutionary dynamics of NSCLC. Although tumor stage positively correlated with the proportion of nonsynonymous mutations, this correlation was not statistically significant (P=0.23) (Fig. S3D). Furthermore, there was no significant association between tumor stage and the number of mutations, mutated genes, or the proportion of COSMIC mutations (Fig. S3A–C). Additionally, there were no significant differences between metastasis and non-metastasis patients in terms of the number of mutations (P=0.27) and mutated genes (P=0.51), or the proportion of COSMIC (P=0.95) and nonsynonymous mutations (P=0.55) (Fig. S4). Notably, across all patients, most tumor cells harbored nonsynonymous gene mutations such as *MUC12*, *KMT2C*, *PDE4DIP*, and *NOTCH2NL*, with significant functional impact scores based on SIFT score ( < 0.05) [46] and PolyPhen (*p* > 0.85) [47], suggesting their important roles in NSCLC progression (Fig. S5). For example, *KMT2C* deficiency has been reported as a major driver for small cell lung cancer metastasis [48].

### Single-cell phylogenetics

**Fig. 2:**
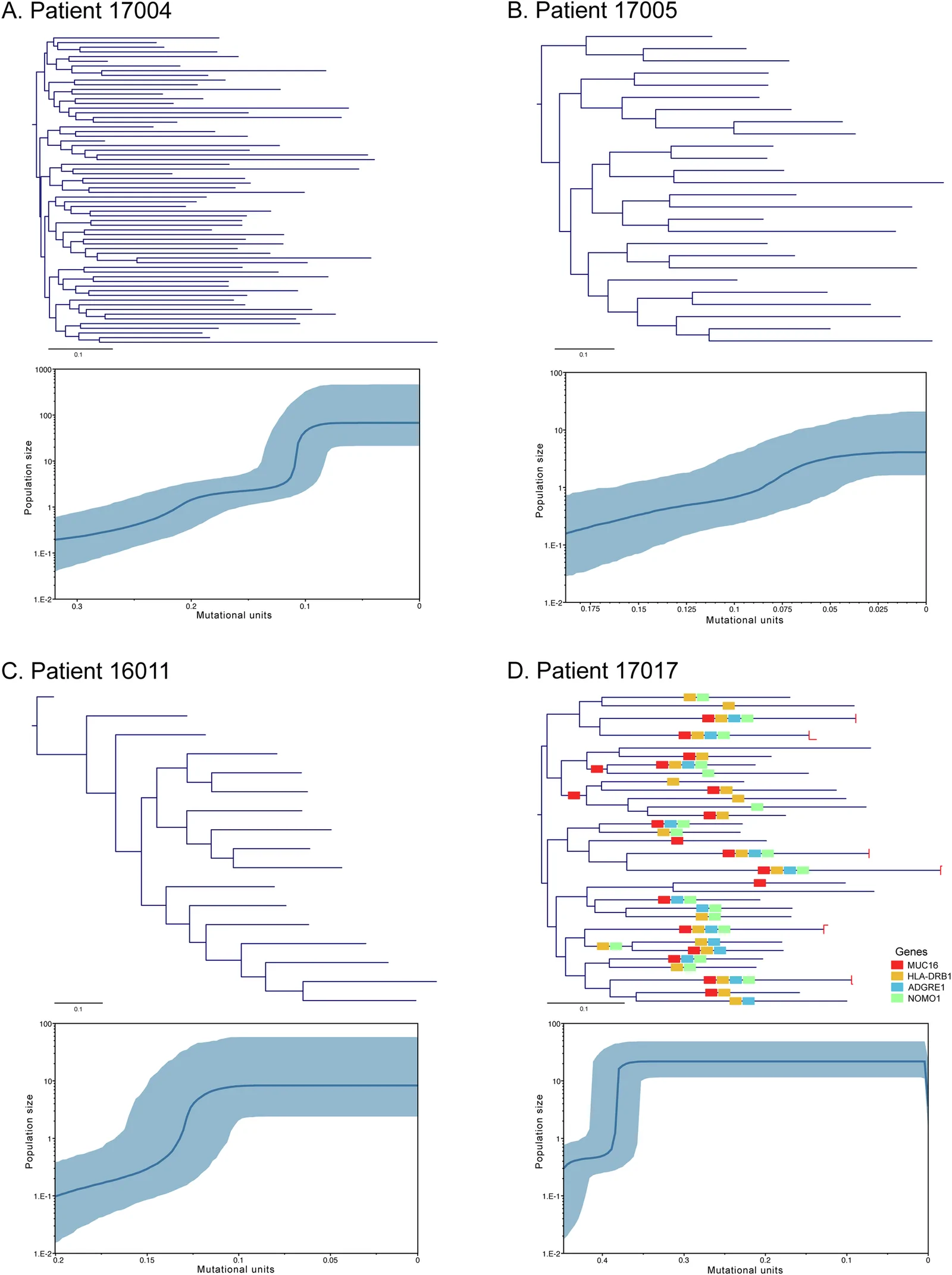
Phylogenetic trees and skyline plots based on the single-cell WES data of four NSCLC patients exemplify four phases of the proposed NSCLC evolutionary model. **A** Patient 17004 illustrates a phase of rapid expansion via Big Bang-like growth. **B** Patient 17005 illustrates a phase of stasis or tumor dormancy via neutral evolution. **C** Patient 16011 illustrates a phase of gradualism, with relevant subclades under selective-sweep-like patterns. **D** Patient 17017 illustrates a phase of punctuated evolution, with a recent bottleneck event on the skyline plot. Red branches denote cells that may potentially evolve into DCC-like cells or dissemination-prone subclones. Non-synonymous mutations were mapped to the tree, while four mutated genes were identified and shared across all internal branches of these pairs of cells. The phylogenetic trees were rooted using their corresponding normal bulk sample, which was subsequently removed and not shown on the trees. The scale bar represents the expected number of substitutions per site. In the skyline plot, the medians of the estimates are indicated by the blue line, and the 95% credibility intervals are shown by the transparent blue areas.

The single-cell phylogenetic analysis for each patient was performed with filtered SNVs using CellPhy [37], leveraging its error and diploid genotype models. The phylogenetic analyses for all 11 NSCLC patients recovered fully bifurcating trees, with most branches receiving reasonable bootstrap support values (>50%). Notably, these phylogenetic trees showed an association with the progression of tumor stage (Figs. 2 and S6): (i) In Stage IA, there is a noticeable pattern where the external branch lengths are much longer than the internal branches (Figs. 2A, B and S6A–D), particularly in Patients 17004 and 17028, who exhibited exceptionally high external to internal (EI) ratio exceeding 8 and well-balanced phylogenetic trees (Figs. 2A and S6A, B); (ii) In Stage IB through IIA, the ratios between the external branch lengths and the internal branch lengths decrease, coupled with quite imbalanced trees characterized by ladder-like phylogenies in related clades (the exception is Patient 17011), in comparison to Stage IA (Figs. 2C and S6E–H); and (iii) In Stage IIB and advanced Stage IV, all patients presented with metastatic tumors, especially in Patients 17017 and 17029, who died within two years after surgery. Except for Patient 18001 with a ladder-like phylogeny, the other two patients exhibited well-balanced phylogenetic trees, and notably, several pairs of cells (cherry tips) with very short external branch lengths and considerably long internal branch lengths (very low EI-ratio) were identified (Figs. 2D and S6I–K). This suggests that these pairs of cells are closely related to each other but differ significantly from other cells on the tree. Furthermore, after mapping the non-synonymous mutations onto the branches of the phylogenetic trees for Patients 17017 and 17029 (Figs. 2D and  S6J, K), we found four mutated genes (*MUC16*, *HLA-DRB1*, *ADGRE1*, and *NOMO1*) shared across all internal branches of pairs of cells with very low EI-ratio, in both patients (although each patient possessed their own specific SNVs within these genes; Table 2). These four genes have been found to be related to tumor cell adhesion and proliferation, as well as immune surveillance and evasion, thereby potentially facilitating tumor metastasis [49–52] (Table 2). Thus, these four genes may represent candidate prognostic biomarkers or therapeutic targets in NSCLC, warranting further investigation in larger cohorts, particularly given their reported relevance in other cancer types, such as *MUC16* in ovarian cancer [53], *HLA-DRB1* in cutaneous melanoma [54], and *ADGRE1* in colorectal cancer [55]. In addition, several other terminal branches in those two patients also exhibited these four mutated genes, suggesting the possibility that not all such cells (i.e., pairs of cells with very low EI-ratio) were collected during sampling (Figs. 2D and  S6J, K).

**Table 2:** The non-synonymous mutations affecting genes shared across all internal branches of paired potential disseminated cancer cells (DCCs) in patients 17017 and 17029.

Gene	SNP ID		Functions related to metastasis
	Patient-17017	Patient-17029	
*MUC16*	rs2547072, rs2591591, rs2591593, rs17000836	rs1862460, rs2591597, rs11085776, rs11085777, rs12611293, rs56307190, rs73011014	*MUC16* (CA125), the largest member of the transmembrane mucin family, which plays a critical role in cancer cell adhesion [88], proliferation [89] and invasion [90], immune response [49], and metastasis [91, 92].
*HLA-DRB1*	rs1064592, rs56158521, rs111739605	rs707953, rs17884945	*HLA-DRB1* encodes the beta chain of the major histocompatibility complex class II, pivotal for immune recognition and response [93, 94]. This gene is significantly implicated in the evasion of DCCs from immunosurveillance [50], thereby facilitating tumor metastasis.
*ADGRE1*	rs330877	rs457857	*ADGRE1* (EMR1), a member of the adhesion G protein-coupled receptor family, has been found to be associated with promoting lymph node metastasis and poor prognosis [55] (e.g., in colon cancer), particularly through its interaction with tumor-associated macrophages [52].
*NOMO1*	rs1062412	rs62038492	*NOMO1* (Nodal modulator 1), an important gene involved in the regulation of the Nodal signaling pathway [95] and may act as a tumor suppressor gene [51], which can increase the invasive and metastatic capabilities of tumor cells by modulating Nodal expression [96, 97].

### Phylogenetic tree statistics and clustering

**Fig. 3:**
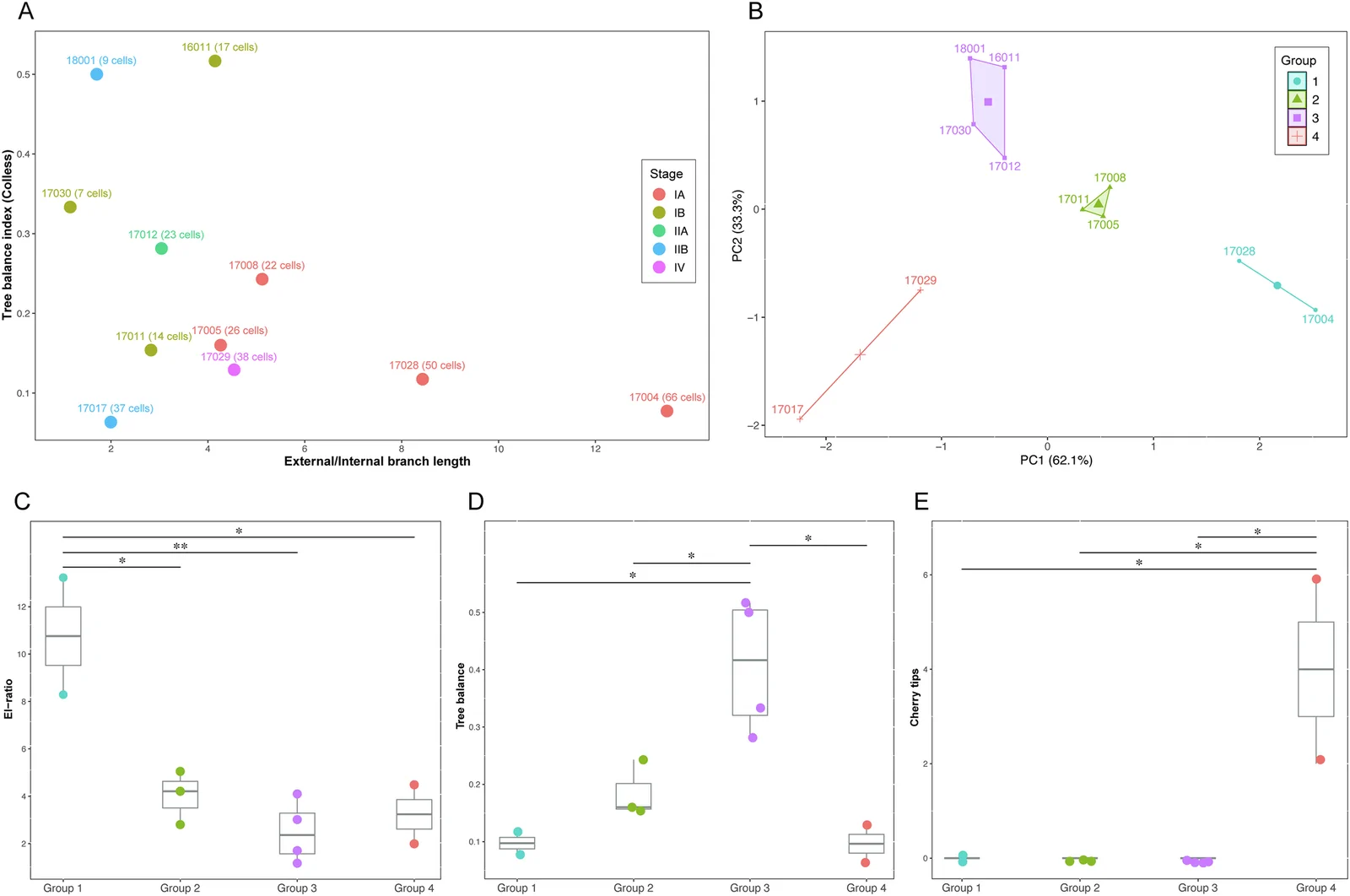
Phylogenetic tree statistics of 11 NSCLC patients. **A** The scatter plot shows the relationship between different stages (colors) of NSCLC and tree statistics, where the tree balance index of Colless is plotted against the ratio of external to internal branch lengths (EI-ratio) across the phylogenetic trees of all 11 patients. **B** Principal component analysis (PCA) of patients based on EI-ratio, Colless index, and number of Cherry tips with short external and long internal branches. This plot displays the first two principal components, with colors indicating four distinct groupings. The boxplots present the EI-ratio (**C**), Colless index (**D**), and number of these Cherry tips (**E**) for each patient across the four groupings.

A complementary approach to depict the modes of tumor evolution involves analyzing the phylogenetic tree shapes [56]. However, it should be noted that similar phylogenetic patterns can arise from different evolutionary processes and/or technical factors (e.g., sampling effects) [34]. To further investigate the relationship between different stages of tumor evolution and tree shapes, three tree balance indices (Colless [57], Cophenetic [58], and scaled Sackin) were individually plotted against the ratio of external versus internal branch lengths (EI-ratio) for all 11 patients’ phylogenetic trees (Figs. 3A and  S7). These tree statistics plots were largely congruent. We further performed principal component analysis (PCA) incorporating the cherry tips (i.e., a clade or cluster of a pair of cells with short external branches, at the end of a long internal branch), which revealed clustering of our patients into four groups (Fig. 3B). Because patient grouping may be influenced by the choice of tree shape metrics and the number of single cells sampled per patient, we interpret these groupings cautiously as exploratory groups. Additionally, to assess whether these grouping patterns were influenced by uneven cell numbers across patients, we performed a downsampling sensitivity analysis, which yielded a similar grouping pattern (Fig. S8). Nevertheless, further validation in larger single-cell WES NSCLC cohorts with more uniform cell numbers across patients will be important.

The first group includes Patients 17004 and 17028 (Stage IA) with the highest EI-ratio and lower tree balance index (representing a more balanced tree) (Figs. 3A–D and  S6A, B). This pattern is compatible with rapid population expansion without clear evidence of strong selection (Box 2), implying that these tumors are at an early phase of tumor progression.

The second group, including Patients 17005, 17008 and 17011 (Stage IA and IB), may be at a later phase of early tumor progression, with a relatively high EI-ratio and higher tree balance index compared to the first group (Figs. 3A–D and  S6C–E). Plausibly, these patients may have experienced a certain level of selection, perhaps a succession of selective sweeps, leading to a less balanced tree and a growth rate that may not be as rapid as observed in the previous phase.

The third group contains Patients 16011, 17030, 17012, and 18001 (Stage IB, IIA, and IIB), considered as a later phase of tumor progression, with the highest tree balance index (representing a more imbalanced tree) and lower EI-ratio (Figs. 3A–D and  S6F–I). Moreover, the presence of ladder-like phylogenies in those relative clades may be compatible with strong directional selection and selective sweeps (Box 2), potentially reflecting immune-mediated selection [17], or replacement of reproductively fitter variants.

The fourth group consists of Patients 17017 and 17029 (Stage IIB and IV, with metastasis and subsequently died), representing advanced stage disease, exhibiting a relatively low tree balance index with a low EI-ratio, largely due to the presence of several clades of cherry tips—pairs of cells with a very low EI-ratio (Figs. 3 and  S6J, K). The presence of closely related pairs of cells, picked up in the sample of cells sequenced, raises the possibility that these cells may represent emerging subclonal variants with potential escape from immune control, and/or the emergence of disseminated cancer cells (DCCs)-like cells with the potential for clonal expansion (i.e., invading vessels and seeding distant metastases [59]). However, this interpretation requires further orthogonal validation, such as spatial sampling, copy-number profiles, and functional assays.

**Fig. 4:**
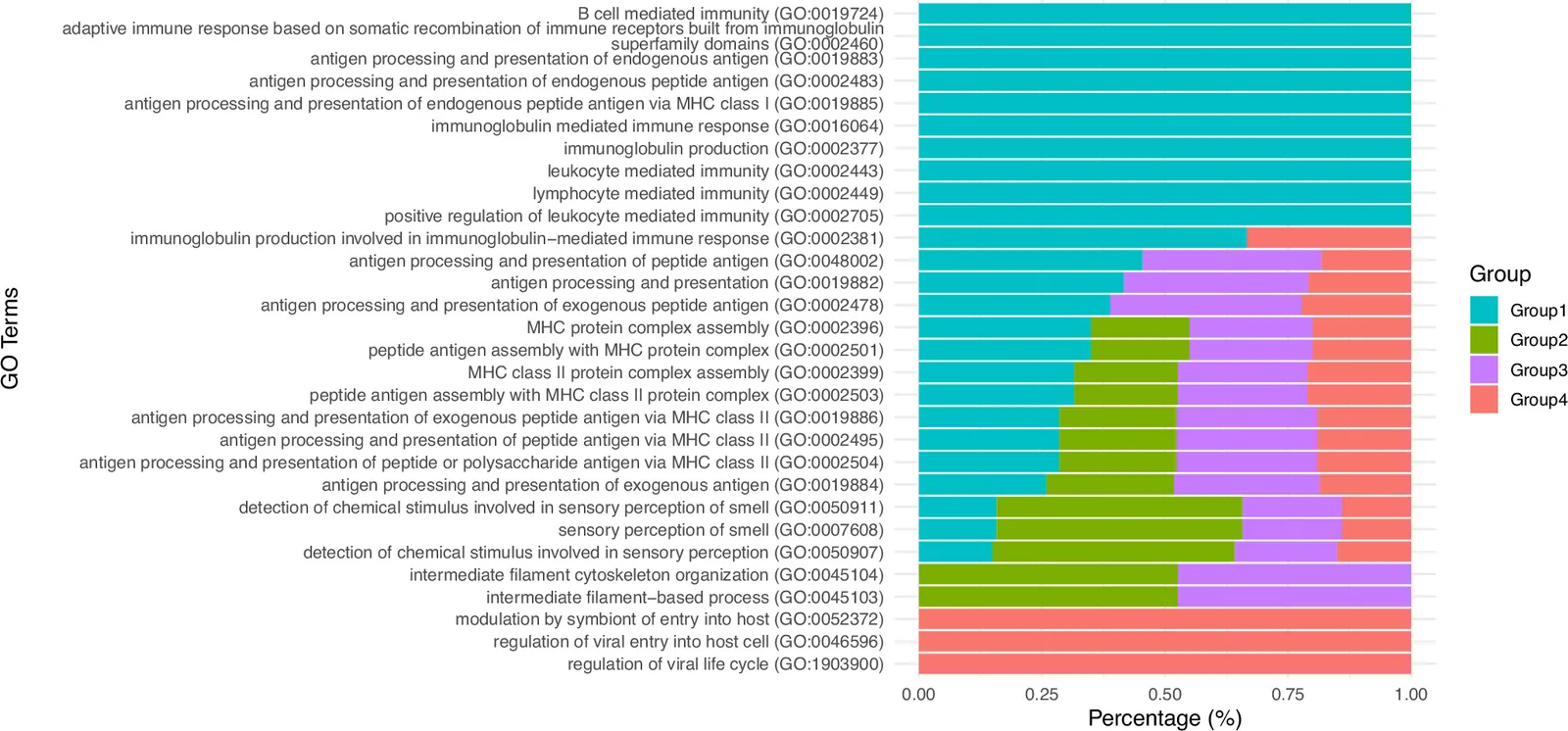
Gene Ontology enrichment analysis of mutated genes across patient groups. Gene Ontology (GO) enrichment analysis was performed using the R package clusterProfiler [87], based on nonsynonymous gene mutations. The top 30 GO terms in biological process across all patient groups were selected based on the minimum adjusted p-value.

Furthermore, Gene Ontology (GO) analyses of nonsynonymous gene mutations revealed differences in enriched biological processes across the four groups (Fig. 4). These GO term enrichments are suggestive of potential biological processes involved, although further validation is required in larger cohorts with matched functional or transcriptomic analyses. Several GO terms enriched in the first group were related to immune surveillance, including antigen processing and presentation, B cell mediated immunity, and immunoglobulin mediated immune response, raising the possibility that immune recognition and elimination may remain intact during the early phase of tumor progression. All four groups shared a core set of GO terms, including MHC protein complex assembly, peptide antigen presentation via MHC class II, and, interestingly, olfactory perception. In particular, analysis of the second group identified a large proportion of biological processes related to sensory perception of smell and associated chemical stimulus detection (GO:0007608, GO:0050907, and GO:0050911), potentially reflecting epigenetic changes in response to tumor development or treatment-induced alterations. For example, a previous study reported that chemotherapy could affect the perception of smell in breast cancer patients [60]. Notably, intermediate filament-related GO terms (GO:0045103 and GO:0045104) were enriched in Groups 2 and 3, which may be relevant to tumor development and invasion, as involvement in epithelial-to-mesenchymal transition and cell motility may enhance the invasive potential during tumor progression [61]. Remarkably, the fourth group exhibited enrichment in virus-related biological processes, including modulation by symbiont of entry into host, regulation of viral entry into host cell, and regulation of viral life cycle (Fig. 4). These findings raise the possibility that these advanced-stage tumors may exploit “viral mimicry” mechanisms to escape immune surveillance, thereby potentially promoting immune evasion and facilitating metastasis. Although “viral mimicry” is typically associated with the activation of antiviral response, a recent study demonstrates that chronic interferon induction can conversely increase immune tolerance and diminish immunogenicity [62].

In addition, each patient group harbored a set of mutated genes shared within the group (Fig. S5), except for Group 3, likely due to the small number of single cells (≤ 10) from two of its patients (Patients 17030 and 18001). These group-specific nonsynonymous mutations provide additional support for the clustering of these patients into four different groups, although their statistical robustness is limited by the small number of patients in each group.

### Single-cell phylodynamics

Phylodynamics offers a powerful method to explore tumor population dynamics and evolutionary processes [32], especially when direct monitoring of the progression of the patient with NSCLC is impractical. To address this issue and examine the pattern of evolutionary history for the four groups identified above, Bayesian coalescent skyline plots, coupled with the diploid genotype error model (implemented in BEAST2 [38]), were reconstructed for each patient using filtered SNVs (Figs. 2 and  S6).

The skyline plot results are overall consistent with the findings of the phylogenetic analysis and the analysis of tree shape parameters (Figs. 2 and S6). Patients 17004 and 17028 with early Stage IA exhibited nearly identical evolutionary trajectories, characterized by a recent rapid expansion in population size after an extended period of stable and gradual growth (Figs. 2A and  S6A, B). In addition, the median historical values of effective population size suggest the possibility of another plateau followed by expansion during this extended phase. This implies that these patients may have undergone a preceding undetected stage (e.g., Stage 0) prior to the recent clonal expansion.

Patients 17005, 17008, and 17011 with Stage IA and Stage IB showed similar tumor population dynamics, with a smooth and slow growth in population size (Figs. 2B and  S6C–E). This trend could potentially be attributed to the immunological response in eliminating malignant cells [63].

Patients 16011, 17030, 17012, and 18001 had stage IB–IIB disease and displayed variable patterns in their skyline plots, but all showed a prolonged phase of constant effective population size in their tumor cells (Figs. 2C and S6F–I). For example, in Patient 16011, there was an initial increase in the effective population size, which then entered a phase of population stability over a long period (Fig. 2C). When we examined the associated phylogenies, we observed ladder-like subclades potentially indicating the possibility that selective sweeps may be operating on some lineages in the tumor. One possible explanation is that the immune system prevents further tumor progression [6]. Over this phase, immune pressure may also select cancer cells that have acquired advantageous mutations that are able to evade elimination, a process commonly referred to as immune escape [17, 59] (Fig. 1).

**Fig. 5:**
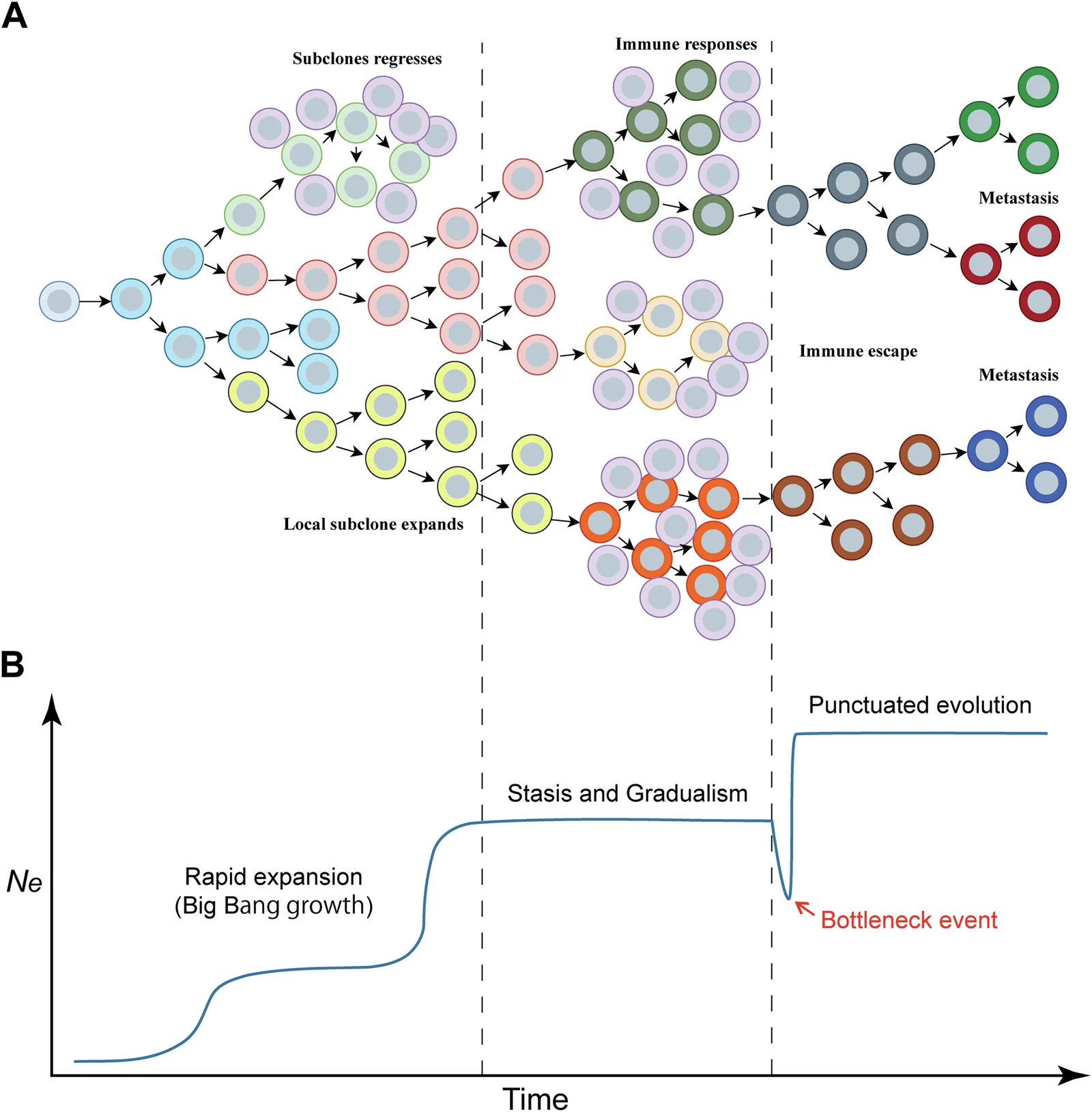
Proposed model of NSCLC progression. **A** Tumor architecture of proposed model. Once cancer is initiated, a rapid expansion phase ensues, via Big Bang-like growth. This phase is subsequently followed by a prolonged plateau period of stasis and then gradualism. This is succeeded by a sudden drop in population size, with a bottleneck event wherein multiple subclones acquire invasive growth capabilities through punctuated evolution. Following this, a phase of rapid expansion in population size is anticipated after these DCC-like cells invade a vessel and seed distant metastases. The cell annotation is the same as for Fig. 1. **B** Corresponding population dynamics plot. The x-axis is the evolutionary time, and the y-axis is the expected effective population size.

Patients 17017 and 17029 with Stage IIB and beyond showed a decline in effective population size after a plateau period, due to the presence of several pairs of cells with distinctly short external and long internal branch lengths (Figs. 2D and  S6J, K). Moreover, the positioning of these several cherry tips as polyphyletic groups may indicate that multiple lineages possess the capability for clonal expansion. These multiple subclones may individually contribute to episodes of tumor relapse and metastasis, serving as the bottleneck of multiclonal invasion, which is broadly consistent with the tumor invasion model proposed by Casasent et al. [64]. Particularly, Patient 17017 demonstrated an evolutionary history that encompassed the evolutionary patterns of nearly all previous stages, starting with a sharp increase in population size, followed by a prolonged plateau, and then a sudden dip in population size at the most recent period (Fig. 2D). This pattern is consistent with, and encompasses, the evolutionary trajectories of these earlier-stage NSCLC patients, but also includes a recent population bottleneck occurring in the advanced stage. Furthermore, considering the potential for clonal expansion in these multiple subclones, we anticipate a rapid expansion phase may follow this bottleneck (Fig. 5), with the requirement of sampling additional tumor cells from invaded vessels and related metastatic tumors.

### NSCLC evolutionary model

Tumor evolutionary models, which describe the initiation, progression, and dissemination of cancer, are crucial for interpreting tumor growth dynamics and could play an important role in the diagnosis, prognosis, and treatment decisions of cancer patients [13, 14, 41, 56]. Several competing models, including linear, branching, neutral and punctuated evolution (see Box 1) have been adapted to understand tumor dynamics [41, 65]. Additionally, the Big Bang model of human colorectal tumor development posits that a single clonal expansion occurs early after cancer initiation, followed by effectively neutral evolution [13, 56]. However, studies reveal that different tumor types exhibit different evolutionary dynamics, with the possibility of multiple models operating at different phases of progression or simultaneously during tumor evolution [41, 56, 66]. Subsequently, a hybrid model of “punctuated equilibrium in cancer” [14] was proposed, in which tumor populations undergo a series of different phases: stasis, gradualism, and punctuation (Box 1).

Here, we take advantage of single-cell phylogenetics and phylodynamics to explore and describe the evolutionary dynamics of NSCLC, focusing on the primary tumor and its early stages in the hopes of finding new ways to manage this disease. Our results are partially congruent with Nowell’s model, which allows the branching evolution of subclones, suggesting that subclones evolve largely independently and in parallel [6]. However, our data are consistent with a mixed model in NSCLC evolution and allow us to propose a hypothesis-generating framework comprising the following phases (Fig. 5): (i) Once cancer is initiated, a single clonal expansion occurs via Big Bang-like growth. This is characterized by quite balanced phylogenetic trees with extremely short internal branches and notably long terminal branches (high EI-ratio), which may indicate tumor cells with significant degrees of divergence, leading to a high level of intratumoral heterogeneity (ITH) (Fig. 2A). This is consistent with previous TRACERx studies, which demonstrated pervasive ITH and early clonal diversification in primary NSCLC tumors [15, 18]. Additionally, our skyline plots suggest the possibility of another plateau followed by an expansion phase, possibly a consequence of selective pressures (e.g., immune response) that regulate tumor growth, but which also allow some selectively advantageous mutant subclones to expand while others may become extinct, regress, or remain dormant (Fig. 5). (ii) Subsequently, a relatively long period of stasis or tumor dormancy (observed as a period of stability in effective population size on the skyline plot) potentially indicates a period of neutral evolution, with the phylogenetic trees displaying shorter terminal branches and longer internal branches (Fig. 2B), indicating tumor cells within each subclone have more similarities than in the previous phase. (iii) At a certain point, continuous and strong selective pressures act upon the tumor cell population, leading to gradualism, as evidenced by selective-sweep-like patterns in relevant subclades with imbalanced tree structures (Fig. 2C). This pattern is compatible with TRACERx findings of subclonal selection in NSCLC, which showed that large subclonal expansions are associated with positive subclonal selection [18]. Meanwhile, other subclones can co-exist without being outcompeted by the subclones undergoing selective sweeps. (iv) These selectively advantageous subclones may give rise to several pairs of cells with long internal branches and notably short terminal branches (Fig. 2D), which is a key feature of punctuated evolution. These pairs of cells harbor specific mutated genes (i.e., *MUC16*, *HLA-DRB1*, *ADGRE1*, and *NOMO1*, as identified in our patients) previously implicated in tumor immune invasion, cell adhesion, and proliferation, raising the possibility that they may contribute to tumor evasion (e.g., immune escape) and metastatic progression [49–52] (Table 2). Thus, these cells may have the potential to evolve into DCC-like or dissemination-prone subclones. However, these gene mutations alone do not establish metastatic competence, and this interpretation requires further validation using related metastatic samples (e.g., invaded vessel samples), ideally integrated with robust single-cell somatic copy-number alteration analysis. Moreover, the polyphyletic placement of these cherry tips may suggest that multiple subclones have acquired invasive growth capability, which is consistent with TRACERx findings that NSCLC metastases can arise through polyclonal dissemination and branched metastatic seeding [15]. This may lead to a sudden drop in population size, resulting in a bottleneck on the skyline plot (Fig. 2D). If these DCC-like cells subsequently invade vessels and seed distant metastases, a rapid expansion phase is expected following this bottleneck event (Fig. 5). Given that these cells harboring related mutations in the above four genes in patients who had metastasis and subsequently died (i.e., Patients 17017 and 17029), and findings from studies involving these DCC-like cells [67], the presence of such cherry tips may represent a candidate phylogenetic feature associated with aggressive disease, although larger cohorts with related metastatic samples will be required to evaluate its prognostic value. In addition, the latter three phases of this new model are fundamentally consistent with the process described by the hybrid model of punctuated equilibrium [14].

This proposed NSCLC evolutionary model is a hypothesis that is based on the development of NSCLC tumors through various stages of evolution and requires further refinement and validation in larger single-cell WES NSCLC cohorts with matched metastatic lesions and longitudinal data. However, we do not preclude the possibility of abrupt progressions in tumor stages, such as a leap from Stage IA (e.g., Patient 17004) directly to Stage IIB (e.g., Patient 17017). Furthermore, we also noticed that the existing staging system (i.e., the TNM system), which classifies tumors based on size, lymph node status, and distant sites of metastasis, does not fully encapsulate the evolutionary dynamics observed in single-cell phylogenetics and phylodynamics. For example, before spreading to lymph nodes, tumors in stages IA, IB, and IIA are clinically differentiated by increasing tumor size. However, our findings suggest that these tumors may undergo significantly different evolutionary processes. Therefore, our results demonstrate that the evolutionary dynamics and phylogenetic insights gained from single-cell phylodynamics and phylogenetics offer an alternative understanding of NSCLC progression, shedding light on a new way to more effectively monitor and predict cancer progression, treatment response, and survival.

## Materials and methods

### Patients and samples

This study was approved by the Duke University Health System Institutional Review Board, with all patients providing written informed consent for the utilization of their tissues in this research. For a complete ethics statement, please refer to Li et al. [68]. A total of 11 sequential NSCLC patients were selected for this study, with their demographic and tumor-related information provided in Table 1. These patients were staged according to the eighth edition of the American Joint Committee on Cancer and received surgical treatment from 7 April 2016 to 20 Jan 2018. Among these patients, eight individuals with early-stage NSCLC had a recurrence-free period after resection, ranging from 14 to 45 months. The duration endpoint for the non-metastatic designation in each of these cases was the last clinical examination following their initial diagnosis. The other three patients presented with metastasis at the time of diagnosis, with two of them having metastasis to lymph nodes, while one patient had lung metastasis. Furthermore, based on the histological examination, seven of 11 patients were diagnosed with adenocarcinoma, two patients were classified as having squamous cell carcinoma, one patient had large cell carcinoma, and one patient was diagnosed with spindle cell carcinoma, a rare type of NSCLC [69].

### Isolation of tumor cells from NSCLC tumor tissue

Freshly resected lung tumor tissue was dissociated into a single-cell suspension using a Tumor Dissociation kit (Miltenyi Biotec GmbH, Germany), following the manufacturer’s instructions. The suspension was filtered through a 70 µm nylon screen to remove macroscopic debris. Subsequently, lymphocytes, fibroblasts, and endothelial cells were eliminated using the Miltenyi Tumor Cell Isolation kit. The recovered tumor cells were resuspended in Bambanker (Lymphotec Inc., Tokyo, Japan) cell freezing medium and frozen at −80 °C. After thawing, cells were stained with mouse anti-human CD45RA PE-Cy7 (clone L48, BD Biosciences, San Jose, CA) and Aqua Vital Dye (Invitrogen, Carlsbad, CA). Finally, cells were sorted individually into the wells of the 96-well PCR plate under fluorescence-activated cell sorting (FACS), which was conducted with a BD FACSDiva with 70 psi sort pressure and a 70 µm nozzle. These sorted single cells were gated as alive/CD45^neg^ and then sealed with the plate sealing tape before being stored in a −80 °C freezer until DNA amplification.

### Single-cell whole genome amplification

All selected single cells were subjected to whole genome amplification (WGA) using the REPLI-g Single Cell kit (Qiagen, Hilden, Germany) following the manufacturer’s instructions. The effectiveness of WGA was verified by PCR amplification of a region of the kinesin light chain 2 gene (KCL2), followed by agarose gel electrophoresis.

### Normal bulk DNA extraction

Normal bulk DNA was isolated from normal lung tissues obtained during surgery and stored at −80 °C, or from FFPE sections. The extraction was performed using a QIAamp Fast DNA Tissue kit for fresh tissues or a QIAamp DNA FFPE Tissue kit for FFPE sections (both from Qiagen, Hilden, Germany), respectively, following the manufacturer’s guidelines.

### Whole exome sequencing

Single-cell whole-genome amplified DNA from tumor cells and bulk DNA from normal tissues were initially processed with KAPA Beads (Roche; Basel, Switzerland) to eliminate any remaining buffer or EDTA. Subsequently, we constructed sequencing libraries with the KAPA HyperPLUS kit (Roche) using the Hamilton NGS Star liquid handler (Hamilton Robotics, Cary, NC, USA), following the manufacturer’s protocol. In brief, samples were processed through enzymatic fragmentation, followed by end repair, A-tailing, and ligation with sample-specific barcodes (Integrated DNA Technologies, Coralville, IA, USA). After library preparation and amplification, the libraries were normalized and pooled, ready for exome capture. Exome enrichment captures were performed with the IDT xGen Exome Research panel on the Hamilton NGS Star liquid handler in accordance with the rapid capture protocol. Finally, the captured libraries were quantified by quantitative polymerase chain reaction (qPCR) with KAPA Quant (Roche) and qualified using the AATI Fragment Analyzer (Agilent Technologies, Santa Clara, CA, USA). The qualified libraries were then sequenced using the Illumina NovaSeq 6000 sequencer (Illumina Inc., San Diego, CA, USA).

### Whole exome sequencing data pre-processing

All FASTQ files from whole exome sequencing (WES) were mapped to the human reference genome (hs37d5) using Burrows-Wheeler Aligner (BWA) with the BWA-MEM algorithm [70] as implemented in the Sentieon Genomics bioinformatics suite [71]. Both duplicated reads in the alignment and reads with mapping quality scores below 40 were removed using SAMtools [72]. Then, Genome Analysis Toolkit (GATK) [73], implemented in Sentieon Genomics [71], was employed to perform indel realignment and base quality score recalibration based on known indels from the 1000 Genomes Project and SNPs from dbSNP [74].

### Filtering single-cell samples

Single-cell samples that were not of sufficiently high quality for single-nucleotide variant (SNV) detection and subsequent analyses were excluded. The average coverage depth and breadth of the exome target region for each single-cell sample were calculated using SAMtools [72], and the visualization of the cumulative coverage was performed with BEDTools [75] and plotted in R [76]. For each patient, single-cell samples with an average coverage depth below 10× and outliers in the breadth (i.e., breadth less than 15% of the target region with a depth greater than 10×) were removed from the analysis (Table S1). Consequently, 42 single-cell samples with low coverage depth and breadth were excluded (Fig. S9).

### SNV calling in single-cell samples

Two robust and widely used single-cell-specific variant callers, Monovar [24] and SCIΦ [26], were employed for detecting and genotyping SNVs under default parameters for each patient in our single-cell data sets. Firstly, we identified SNVs only in single tumor cells for several reasons: (i) post-zygotic mutations are frequently shared among many tissues, making it difficult to identify a clear normal matched bulk tissue [77]; (ii) normal tissue adjacent to the tumor has been reported to present a unique intermediate state between healthy and tumor [78]; and (iii) those single-cell SNV callers are specifically designed for single-cell data (e.g., Monovar [24]). To further reduce false positives in SNV calling, only the SNVs detected by both callers were retained for subsequent analysis. For each patient, a number of filtering steps were then performed to generate highly confident SNVs by using VCFtools: (i) retain only SNVs within the exome target region and its 100 bp flanking regions; (ii) remove SNVs with more than two alleles; (iii) remove SNVs with mean depth values over all samples less than 10; (iv) assign genotypes as missing if their read depth (DP) less than 5; (v) remove SNVs with minor allele frequency (MAF) less than 0.05; (vi) remove SNVs with variants called in only one cell; (vii) remove Loss of Heterozygosity (LOH) blocks containing five consecutive LOH-like SNPs, where the same ≥50% of single cells showed concordant genotype-supported homozygous allelic imbalance across all five sites (variant allele frequency (VAF) ≤ 0.15 or VAF ≥ 0.85); (viii) retain SNVs only if they could be genotyped in at least 60% of the samples, except for patients 17017 and 18001, for which a minimum of 50% of the samples were retained (Fig. S1). The filtered SNVs were then annotated using ANNOVAR [79].

### Phylogenetic analysis

CellPhy [37], a maximum likelihood framework for inferring single-cell phylogenies that handles diploid genotypes and considers amplification errors and allelic dropouts, was employed to construct the phylogenetic tree for each patient using the genotype likelihood model (${GT}16+{F0}$). To explore the tree space more thoroughly, 50 random and 50 parsimony starting trees were employed. For each patient, the program was run with the default of automatically determining the optimal number of bootstrap replicates and using their corresponding normal bulk sample as the outgroup, which was generated by GATK HaplotypeCaller [80] in GVCF mode and then merged into the single-cell dataset by BCFtools [81]. Moreover, the non-synonymous mutations were mapped onto the branches of the CellPhy tree for Patients 17017 and 17029, who had metastases and died within two years after surgery.

### Tree statistics

The shapes of the phylogenetic trees generated by CellPhy [37] for each patient were evaluated using estimates of tree statistics, which included the ratio of external to internal branch length (EI-ratio) and three tree balance indices (normalized cophenetic [58], corrected Colless [57], and the newly introduced scaled Sackin). The scaled Sackin’s index ${I}_{{scaled}}(T)$ for a given rooted tree T was calculated as follows:

$${I}_{{scaled}}(T)\,=\frac{{I}_{s}\left(T\right)\,-\,\min {I}_{s}\left(n\right)}{\max {I}_{s}\left(n\right)\,-\,\min {I}_{s}\left(n\right)}$$

where ${I}_{s}\left(T\right)$ represents the original Sackin’s index [82] for a given rooted tree T, n denotes the number of leaves in T, $\min {I}_{s}\left(n\right)$ represents the Sackin’s index of the most balanced tree given n, $\max {I}_{s}\left(n\right)$ represents the Sackin’s index of the most unbalanced tree given n. $\min {I}_{s}\left(n\right)$ and $\max {I}_{s}\left(n\right)$ can be computed as follows:

$$\min {I}_{s}\left(n\right)=\,\left\lfloor {\log }_{2}(n)\right\rfloor \, \cdot \, n \, + \, 2 \, \cdot \,\left(n-{2}^{\left\lfloor {\log }_{2}(n)\right\rfloor }\right)$$

and

$$\max {I}_{s}\left(n\right)\,=\,\left({n+1}\atop{2}\right)-1$$

These three tree balance indices range from 0 to 1, where a lower index value indicates a more balanced tree and a higher value indicates a less balanced tree.

Additionally, to explore whether these tree statistics (e.g., EI-ratio) are influenced by systematic bias associated with the varying number of cells among these patients, we randomly sampled 10 cells for each patient and repeated the above analyses, resulting in a similar grouping pattern (Fig. S8).

### Bayesian skyline plot analyses

The coalescent Bayesian skyline method [40] was employed to estimate the past population dynamics from single-cell data for each patient using phylonco [38] in BEAST2 [39]. The GT16 error model [38] for diploid genotypes was applied, coupled with an optimized relaxed clock model [83]. Markov chains were run for 300,000,000 steps for each dataset using the BEAGLE GPU [84] to improve computational performance. The resulting files were used for convergence checks and visualizations of skyline plots using Tracer v1.7.2 [85], with the first 10% of replicates excluded as burn-in, and the effective sample sizes of all parameters were confirmed to be greater than 200. We employ mutation units rather than a chronological time scale because the evolutionary rate varies across different tumor types, stages, and patients [86]. Additionally, the date of diagnosis or sampling usually does not align with the actual initiation of the tumor [20], leaving the root age undetermined.

## Supplementary information

Supplemental information

Table S1

## Data Availability

The raw single-cell whole exome sequencing data for 11 NSCLC patients, along with their corresponding normal bulk tissue sequencing data, have been deposited in the NCBI BioProject repository under accession numbers PRJNA1132716 and PRJNA750918, respectively. The data sets supporting the results, coupled with scripts for all analyses used in this article, are available in the GitHub repository (https://github.com/tengchn/NSCLC_evolution.git).
