# Supplementary material for: Phylogenetic and phylodynamic approaches to understanding the evolution of non-small cell lung cancer using single-cell sequencing
Source: Oncogene. 2026 Sep 24;45(44):4777–90. doi: 10.1038/s41388-026-03997-1 (PMC13713591; doi:10.1038/s41388-026-03997-1)
Supplement: Supplementary file 1 [file 41388_2026_3997_MOESM1_ESM.docx]

Supplemental information for

**Phylogenetic and phylodynamic approaches to understanding the evolution of non-small cell lung cancer using single-cell sequencing**

Teng Li^1,2^*, Xianghan Li^2^, Yiran Zou^2^, Thomas K. F. Wong^2,3^, Michael J. Campa^4^, Elizabeth B. Gottlin^4^, Edward F. Patz Jr^4,5,6^*, Allen Rodrigo^1,2^*

^1^School of Biological Sciences, University of Auckland, Auckland, New Zealand

^2^Research School of Biology, Australian National University, Canberra, ACT, Australia

^3^School of Computing, Australian National University, Canberra, ACT, Australia

^4^Department of Radiology, Duke University Medical Center, Durham, NC, United States

^5^Duke Cancer Institute, Duke University Medical Center, Durham, NC, United States

^6^Department of Pharmacology and Cancer Biology, Duke University Medical Center, Durham, NC, United States

*Corresponding author

Email addresses:

TL: [teng.li@auckland.ac.nz](mailto:teng.li@auckland.ac.nz)

EFP: patz0002@mc.duke.edu

AR: a.rodrigo@auckland.ac.nz

**Figure S1. Overview of genomic heterogeneity in 11 NSCLC Patients.** The top panel shows the number of mutations, mutated genes, COSMIC mutations, and filtered cells, ordered according to the percentage of nonsynonymous mutations across various NSCLC patients. The tumor stage and the percentage of genotype distribution are displayed in the lower panels.

**Figure S2. The percentage of genotype distribution for each of 11 NSCLC patients.** Each bar represents the percentage of different genotypes detected in individual tumor cells for each patient.

**Figure S3.** **Scatter plots displaying the relationships between** **the number of mutations (A), the number of mutated genes (B), the percentage of COSMIC mutations (C),** **and the percentage of nonsynonymous mutations (D) across different tumor stages in NSCLC patients.** The red line indicates the best-fit linear regression. The correlation coefficient ($R^{2}$) and p-value ($P$) of the regression line are presented above each figure.

**Figure S4. Comparison of the number of mutations (A), the number of mutated genes (B), the percentage of COSMIC mutations (C), and the percentage of nonsynonymous mutations (D) between** **metastasis and non-metastasis NSCLC patients.** The p-value ($P$) indicating statistical significance is shown above each plot.

**Figure S5. Single-cell mutation frequency heatmap across all patients.** Mutation frequency per gene across tumor cells was calculated for each patient and color-coded from white to red. Genes with high-risk mutations (SIFT score $<$ 0.05 and PolyPhen $>$ 0.85) are labeled in red. Genes were hierarchically clustered based on their nonsynonymous mutation frequency profiles.

**Figure S6. Phylogenetic trees and skyline plots based on the single-cell WES data of NSCLC patients.** Red branches denote cells that may potentially evolve into disseminated cancer cells. The phylogeny trees were rooted using their corresponding normal bulk sample, which was subsequently removed and not shown on the trees. The scale bar represents the expected number of substitutions per site. In the skyline plot, the medians of the estimates are indicated by the blue line, and the 95% credibility intervals are shown by the transparent blue areas.

**Figure S7. Phylogenetic tree statistics of** **11 NSCLC patients**. The scatter plot shows the relationship between different stages (colors) of NSCLC and tree statistics, where the tree balance indices of Cophenetic (A) and scaled Sackin (B) are plotted against the ratio of external to internal branch lengths across the phylogenetic trees of all 11 patients.

**Figure S8. Phylogenetic tree statistics of randomly sampled 10 cells for each NSCLC patient.** The scatter plot shows the relationship between different stages (colors) of NSCLC and tree statistics, where the tree balance indices of Colless (A), Cophenetic (B), and scaled Sackin (C) are plotted against the ratio of external to internal branch lengths across the phylogenetic trees of all 11 patients.

**Figure S9. Cumulative coverage distribution of exome capture targets for each patient.** Vertical gray lines indicate the depth thresholds (10×, 20×, 50×, 100×), and horizontal gray lines indicate the fraction of target bases (10%, 20%, 50%, 90%). The legend shows each sample ID, total number of mapped reads (in millions), and duplication ratio. Single cells highlighted with a red background in the legend were excluded from downstream analysis.
